# Exercise-induced myocardial edema in master triathletes: Insights from cardiovascular magnetic resonance imaging

**DOI:** 10.3389/fcvm.2022.908619

**Published:** 2022-08-02

**Authors:** Fabrizio Ricci, Giovanni Donato Aquaro, Carlo De Innocentiis, Serena Rossi, Cesare Mantini, Francesca Longo, Mohammed Y. Khanji, Sabina Gallina, Alessandro Pingitore

**Affiliations:** ^1^Department of Neuroscience, Imaging and Clinical Sciences, “G. d’Annunzio” University of Chieti-Pescara, Chieti, Italy; ^2^Department of Clinical Sciences, Clinical Research Center, Lund University, Malmö, Sweden; ^3^MRI Laboratory, Fondazione Toscana G. Monasterio, Pisa, Italy; ^4^Interventional Cath Lab, ASL 2 Abruzzo, Chieti, Italy; ^5^University of Trieste, Trieste, Italy; ^6^Newham University Hospital, Barts Health NHS Trust, London, United Kingdom; ^7^William Harvey Research Institute, NIHR Barts Biomedical Research Centre, Queen Mary University of London, London, United Kingdom; ^8^Barts Heart Centre, St Bartholomew’s Hospital, Barts Health NHS Trust, London, United Kingdom; ^9^Clinical Physiology Institute, CNR, Pisa, Italy

**Keywords:** CMR, deformation imaging, master triathletes, Ironman, athlete’s heart

## Abstract

**Background:**

Strenuous exercise has been associated with functional and structural cardiac changes due to local and systemic inflammatory responses, reflecting oxidative, metabolic, hormonal, and thermal stress, even in healthy individuals. We aimed to assess changes in myocardial structure and function using cardiovascular magnetic resonance (CMR) imaging in master triathletes early after a full-distance Ironman Triathlon race.

**Materials and methods:**

Ten master triathletes (age 45 ± 8 years) underwent CMR within 3 h after a full-distance Ironman Triathlon race (3.8 km swimming, 180 km cycling, and 42.2 km running) completed with a mean time of 12 ± 1 h. All the triathletes had a 30-day follow-up CMR. Cine balanced steady-state free precession, T2-short tau inversion recovery (STIR), tagging, and late gadolinium enhancement (LGE) imaging sequences were performed on a 1.5-T MR scanner. Myocardial edema was defined as a region with increased T2 signal intensity (SI) of at least two SDs above the mean of the normal myocardium. The extent of myocardial edema was expressed as the percentage of left ventricular (LV) mass. Analysis of LV strain and torsion by tissue tagging included the assessment of radial, longitudinal, and circumferential peak systolic strain, rotation, and twist.

**Results:**

Compared with postrace, biventricular volumes, ejection fraction, and LV mass index remained unchanged at 30-day follow-up. Global T2 SI was significantly higher in the postrace CMR (postrace 10.5 ± 6% vs. follow-up 3.9 ± 3.8%, *P* = 0.004) and presented with a relative apical sparing distribution (*P* < 0.001) matched by reduction of radial peak systolic strain of basal segments (*P* = 0.003). Apical rotation and twist were significantly higher immediately after the competition compared with follow-up (*P* < 0.05).

**Conclusion:**

Strenuous exercise in master triathletes is associated with a reversible regional increase in myocardial edema and reduction of radial peak systolic strain, both presenting with a relative apical sparing pattern.

## Introduction

Exercise training and moderate-to-high levels of physical activity have been shown to improve health outcomes and reduce cardiovascular morbidity and mortality (1–3). Despite exercise being an efficacious therapy, there is an incomplete understanding of the entire dose-response relationship. A hypothesis that intense physical training may harm, causing significant myocardial injury, remains unproven (4). Following the initial reports of myocardial fibrosis in athletes about 10 years ago, several studies raised the hypothesis that chronic overload of the cardiovascular system and overtraining may be responsible for myocardial scarring and increased susceptibility to ventricular arrhythmias. However, other studies have outlined different findings questioning the existence of exercise-induced cardiomyopathy (5–7). Cardiovascular MR (CMR) has been used as an imaging technique to assess acute and chronic cardiac changes in endurance athletes, providing information on function and tissue characterization (7–11). CMR data are not entirely concordant about whether intense exercise is harmful to the heart, both in terms of functional abnormalities or myocardial damage. However, the presence of myocardial fibrosis appears to be higher in endurance athletes compared to controls. These data have been confirmed in a recent meta-analysis of CMR studies performed on endurance athletes, showing a higher incidence of myocardial fibrosis by late gadolinium enhancement (LGE) imaging in athletes than in controls, with a predominant non-ischemic LGE pattern at the interventricular insertions (11). In addition, the evidence of myocardial edema, after strenuous exercise, is questionable based on current published data (12–14).

Ironman Triathlon (3.8 km swimming, 180 km cycling, and 42 km running) is a strenuous sports discipline, combining dynamic and static components and causing relevant cardiac adaptations (15–17). We hypothesize that acute changes in cardiac function and myocardial tissue composition occurring after strenuous exercise are fully reversible and there is a relationship between structural and functional changes in the myocardium. Therefore, we used CMR imaging to assess longitudinal changes in myocardial structure and function occurring in master athletes early after completing an Ironmen Triathlon race and a 30-day follow-up.

## Materials and methods

### Study cohort

We enrolled 10 healthy, amateur, Ironman triathletes (9 males, age 45 ± 8 years), with a mean training time of 13 ± 9 years. The inclusion criteria for enrollment were: (i) experience with ultra-triathlon (swimming, cycling, and running) races with at least two completed competitions; (ii) well-trained (>10 h of intense training per week); (iii) no known medical problems; (iv) no cardiovascular risk factors; and (v) no history of performance-enhancing drug use.

The research study design complied with the Declaration of Helsinki and was approved by the local Ethics Committee (Pisa, Italy, protocol number for study acceptance 2,805). After receiving the description of the procedures and potential risks, all the subjects gave their written informed consent.

### Study design

Cardiovascular magnetic resonance was performed twice: (1) within 3 h following completion of the Ironman race (postrace) and (2) 30 days after the race (follow-up). The second CMR scan was performed in resting conditions, asking athletes not to exert physical activity for 2 days preceding the examination.

### Cardiovascular magnetic resonance imaging

Cardiovascular magnetic resonance examinations were performed using a 1.5-T Signa CVI scanner (GE Healthcare, Milwaukee, WI, United States) with a cardiac phased-array 8-channel coil. Left ventricular (LV) and right ventricular (RV) volumes, mass, and function (including assessment of regional wall motion abnormalities) were derived using steady-state free precession [Fast Imaging Employing Steady-State Acquisition (FIESTA)], pulse sequence cine images from the short axis (from atrioventricular valve plane to the apex, 8-mm slice thickness, and no gap), and para-axial views (from the diaphragm to the entire outflow tract, 5-mm slice thickness, and no gap). The following acquisition parameters were applied: 30 phases, 10–25 views per segment depending on heart rate, the number of excitation (NEX) 1, the field of view (FOV) 40 cm, a matrix of 224 × 224, a 45° flip angle, repetition time/echo time (TR/TE) equal to 3.5/1.5 s, and a bandwidth of 125 kHz. T2-STIR images were acquired using triple inversion recovery T2-weighted pulse sequence in short-axis views and two long-axis views (vertical and horizontal long-axis views) using the following parameters: TR = 2 RR, TE 70 ms, FOV 40 cm, phase FOV 1, and matrix 256 × 256. LGE images were acquired 10 min after administering gadolinium-diethylenetriaminepentaacetic acid (Gd-DTPA) (Magnevist, Schering AG) with a dosage of 0.2 mmol/kg. An inversion recovery T1-weighted gradient-echo sequence was used with the following parameters: FOV 40 mm, slice thickness 8 mm, no gap between each slice, TR 4.6 ms, TE 1.3 ms, flip angle 20°, matrix 224 × 224, reconstruction matrix 256 × 256, and an excitation number of 1. The appropriate inversion time was set to null for normal myocardium based on the Look-Locker scout sequence.

Myocardial tagging images for calculation of LV systolic longitudinal, radial, and circumferential strain for basal, middle, and apical segments of the LV were acquired with an electrocardiography-gated, segmented K-space, fast gradient recalled echo pulse sequence with spatial modulation of magnetization to generate a grid tag pattern. Non-selective radiofrequency pulses separated by spatial modulation of magnetization encoding gradients allowed tag separation of 5 mm. Two sets of orthogonally intersecting tag lines were acquired in short-axis views at basal, mid, and apical levels. The number of views per phase was optimized based on the heart rate. The following parameters were used: field of view 40 cm, slice thickness 5 mm, no gap between each slice, repetition time 8 ms, echo time 4.3 ms, flip angle 12°, bandwidth 31 Hz, 30 phases, matrix 256 × 256, reconstruction matrix 256 × 256, and an excitation number of 1.

Using dedicated software (Mass Analysis, MEDIS, Leiden, Netherlands), the following functional parameters were obtained from the short-axis images: RV and LV end-diastolic volume indexed to body surface area, indexed RV and LV end-systolic volume, indexed LV mass, RV and LV ejection fraction (EF), and RV and LV stroke volumes. The indexed RV and LV volumes were compared with the respective age- and sex-specific reference values (18). Myocardial edema was detected as a region with signal intensity > mean + 2 SD of normal myocardium. The extent of myocardial edema was expressed as a % of LV mass. LGE was visually evaluated and measured as a % of LV mass using the conventional mean + 5 SD technique. Tagged CMR images were exported and analyzed with previously validated software (Tagging Tool) (19), and the following LV functional parameters were obtained: global and regional (basal, mid, and apical) radial, longitudinal, and circumferential strain, basal rotation, apical rotation, and twist. All the analyses were performed by two EACVI CMR level 3 certified operators (FR and GA) who were blinded to any clinical information of the athlete and the results of the other examination. Any discrepancies were solved by a third investigator (AP).

### Statistical analysis

Data were expressed as mean ± SD or percentages as appropriate. Differences in immediate postrace vs. resting parameters of the follow-up scans were explored using the paired *t*-test and ANOVA for immediate postrace vs. resting interslice comparison. One-way intraclass correlation coefficients (ICCs) were obtained to assess inter- and intraobserver variability. We considered a 2-sided *p*-value of <0.05 as statistically significant throughout. We ran all the tests with the R statistical software version 3.4.3.^1^

## Results

The demographic and physical characteristics of triathletes are shown in Table 1. Sufficient image quality was obtained in all the participants. Athletes completed the Ironman competition without any cardiovascular symptoms, either during or after the race, in a mean time of 12 ± 1 h.

**TABLE 1:** Characteristics of the study population (*n* = 10).

Age (years)	45 ± 8
Males, n (%)	9 (90)
Height (cm)	174 ± 11
Weight (kg)	70 ± 10
BSA (m^2^)	1.9 ± 0.2

Data are mean ± SD, unless otherwise indicated. BSA, body surface area.

### Ventricular volumes and function

Postrace and resting structural and functional LV and RV parameters are shown in Table 2. All the athletes had normal resting LV and RV functions. Compared with follow-up resting conditions, end-diastolic volumes, stroke volumes, and RV ejection fraction were significantly lower immediately postrace (*P* < 0.05), whereas LV ejection fraction remained unchanged.

**TABLE 2:** Postrace and follow-up CMR imaging parameters.

Variables	Postrace (*n* = 10)	Follow-up (*n* = 10)	*P*-value
Heart rate, bpm	62 ± 5	58 ± 7	NS
LVEDVI, ml/m^2^	87 ± 14	100 ± 16	NS
LVESVI, ml/m^2^	33 ± 9	37 ± 11	NS
LVSVI, ml/m^2^	54 ± 11	63 ± 8	NS
LVEF, %	62 ± 5	63 ± 5	NS
LVCI, L/min/m^2^	3.4 ± 0.5	3.5 ± 0.5	NS
LV mass index, g/m^2^	69 ± 13	68 ± 13	NS
LVSV/ESV	1.8 ± 0.8	1.8 ± 0.6	NS
RVEDVI, ml/m^2^	88 ± 18	98 ± 22	NS
RVESVI, ml/m^2^	36 ± 14	34 ± 14	NS
RVSVI, ml/m^2^	52 ± 12	63 ± 13	NS
RVEF, %	59 ± 6	65 ± 6	NS
RVCI, L/min/m^2^	3.2 ± 0.5	3.5 ± 0.7	NS
RVSV/ESV	1.6 ± 1	2 ± 0.6	NS
Edema, % of LV mass	12 ± 6	3 ± 3	<0.001
Edema, g	16 ± 7	4 ± 4	<0.001
LGE, n (%)	0 (0)	0 (0)	NS

CMR, cardiovascular magnetic resonance; ESV, end-systolic volume; HR, heart rate; bpm, beats per minute; LGE, late gadolinium enhancement; LVCI, left ventricular cardiac index; LVEDVI, left ventricular end-diastolic volume index; LVEF, left ventricular ejection fraction; LVESVI, left ventricular end-systolic volume index; LVSV, left ventricular stroke volume; LVSV/ESV, left ventriculo-arterial coupling; LVSVI, left ventricular stroke volume index; RVCI, right ventricular cardiac index; RVEDVI, right ventricular end-diastolic volume index; RVEF, right ventricular ejection fraction; RVESVI, right ventricular end-systolic volume index; RVSV, right ventricular stroke volume; RVSV/ESV, right ventriculo-arterial coupling; RVSVI, right ventricular stroke volume index.

### Myocardial strain

**FIGURE 1:**
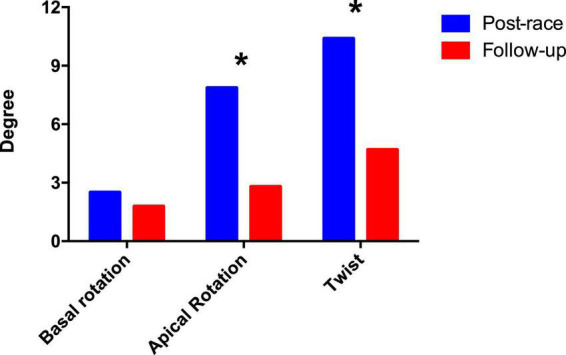
Left ventricular rotation and twist: Postrace vs. 30-day follow-up. *P < 0.05.

**FIGURE 2:**
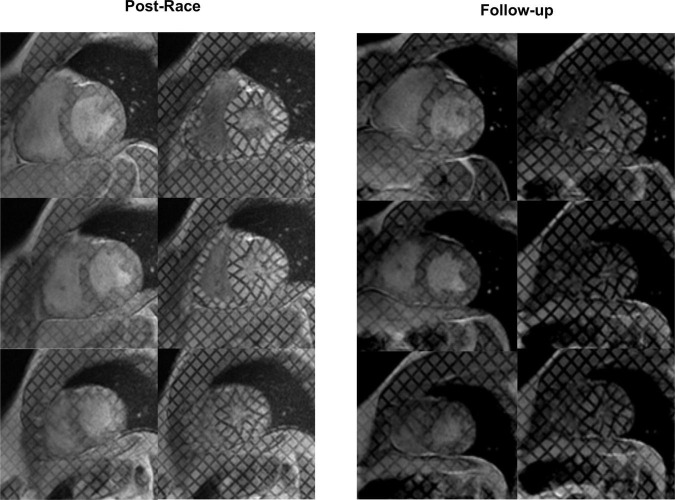
Myocardial tagging: Postrace vs. 30-day follow-up. The radial strain of the basal slice was lower in the postrace than at 30-day follow-up, as demonstrated by the deformation of the tag lines (top rows). Apical twisting was higher in the postrace (left panels) compared with 30-day follow-up (right panels).

**FIGURE 3:**
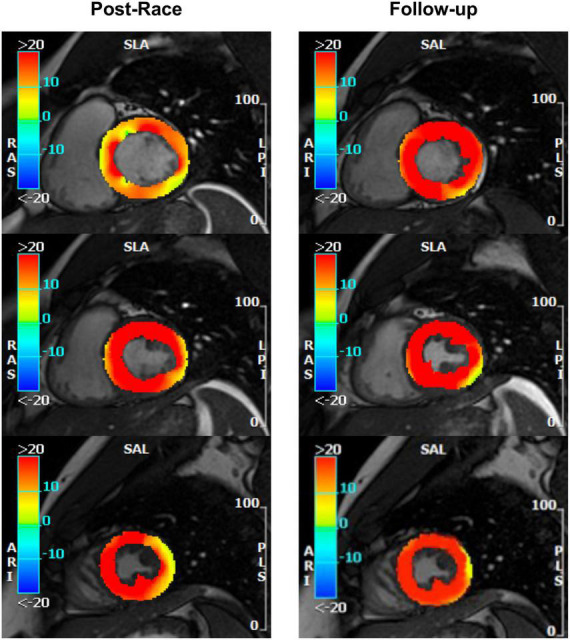
Radial strain: Postrace vs. 30-day follow-up. Feature tracking analysis confirmed tagging evidence of reversible reduction of basal radial strain in the same subject as Figure 2.

Postrace and follow-up myocardial deformation parameters are shown in Table 3. Compared to follow-up, apical rotation and twist significantly increased postrace (*P* < 0.05), whereas LV systolic basal radial strain significantly decreased postrace (*P* < 0.05) (Figures 1–3).

**TABLE 3:** Regional distribution of myocardial edema: Postrace vs. follow-up.

Myocardial edema by T2W-STIR	Postrace	Follow-up	*P*-value^b^	*P*-value*	*P*-value^§^
Basal segments, % of LV mass	5.6 ± 4	1.6 ± 1.1	0.007	NS	<0.001
Mid-cavity segments, % of LV mass	3.8 ± 2	1.3 ± 1.8	0.007		
Apical segments, % of LV mass	1.0 ± 1	0.4 ± 0.7	NS		
Basal segments, g	7 ± 5	1.9 ± 1.3	0.006	NS	<0.001
Mid-cavity segments, g	4.9 ± 2.7	1.5 ± 2.3	0.004		
Apical segments, g	1.4 ± 1.4	0.4 ± 0.7	NS		
Base-to-apex gradient, % of LV mass	4.6 ± 4.3	1.2 ± 0.9	0.026	−	−
Base-to-apex gradient, g	5.6 ± 5.3	1.5 ± 1.1	0.027	−	−

^b^t-test post-race vs. follow-up; *ANOVA inter-slice comparison analysis at follow-up; ^§^ANOVA inter-slice comparison analysis post-race.

### Tissue characterization

**FIGURE 4:**
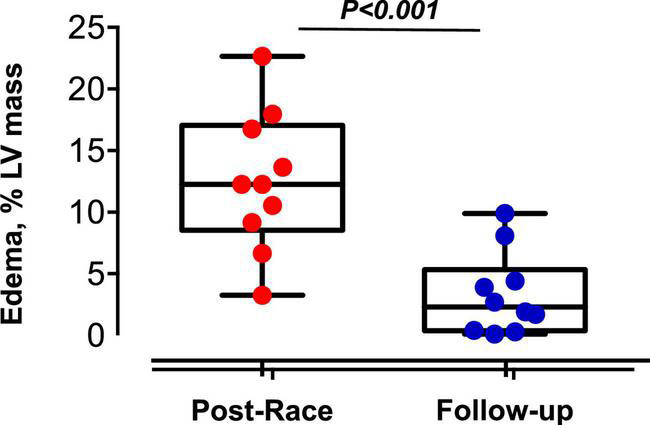
Reversible myocardial edema in master triathletes: Postrace vs. 30-day follow-up.

**FIGURE 5:**
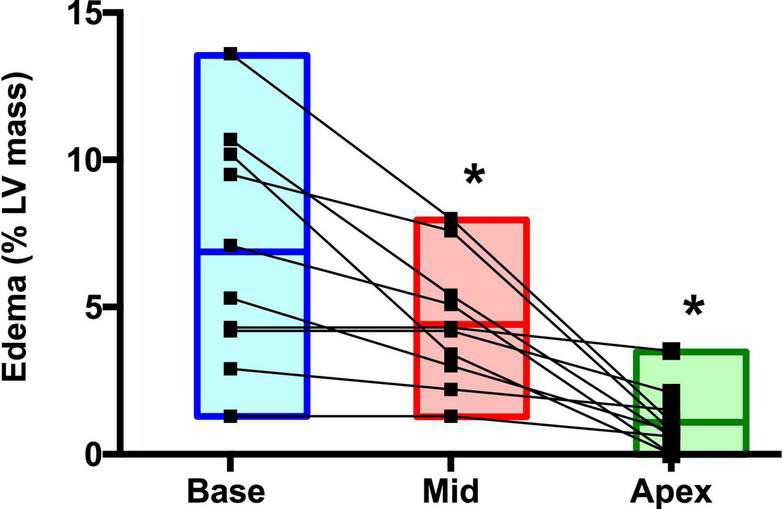
Regional distribution of myocardial edema in the postrace. *P < 0.05.

**FIGURE 6:**
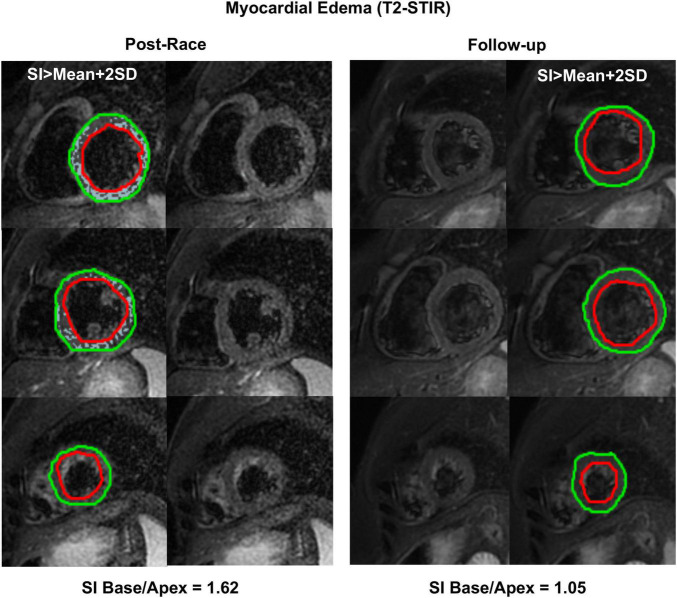
Myocardial edema: Postrace vs. 30-day follow-up. Example of postrace and 30-day follow-up resting T2-STIR images (basal, middle, and apical slices from the complete dataset of short-axis views). Edema was evaluated semi-quantitatively as signal intensity (SI) greater than mean + 2 SD. As shown in the left panels, edema was detected in the postrace acquisition and not at rest (right panels). Moreover, a basal-to-apex gradient was found as the SI ratio basal/apex was greater in postrace than at 30-day follow-up.

**FIGURE 7:**
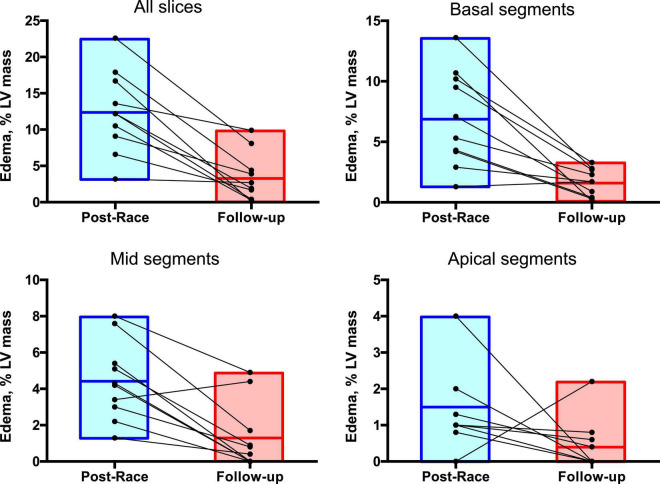
Regional distribution of myocardial edema: Postrace vs. 30-day follow-up. Myocardial edema was detected as a region with signal intensity > mean + 2 SD of normal myocardium. The extent of myocardial edema within each slice was expressed as % of the entire LV mass.

**FIGURE 8:**
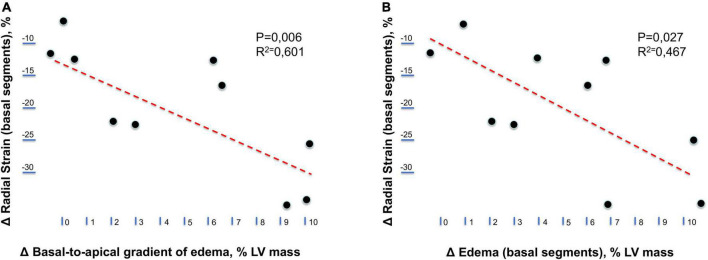
Relationship between longitudinal changes in radial strain and myocardial edema. **(A)** Relationship between temporal changes (postrace vs. follow-up) in the radial strain of left ventricular basal segments and the basal-to-apical gradient of myocardial edema. **(B)** Relationship between temporal changes (postrace vs. follow-up) in the radial strain and myocardial edema of left ventricular basal segments.

Immediate postrace and follow-up assessment of global, basal, middle, and apical LV myocardial edema are shown in Table 4. Compared with follow-up, the extent of myocardial edema was significantly higher immediately after the competition (postrace: 10.5 ± 6% LV mass vs. follow-up: 3.9 ± 3.8% LV mass, *P* = 0.004; Figure 4) and presented with a relative apical sparing distribution (*P* < 0.001) and a basal-to-apical gradient (Figures 5, 6). There was a significant inverse relationship between temporal changes in radial strain and myocardial edema, both tracking the basal-to-apical distribution of edema, or edema localized at basal LV segments (Figures 7, 8). In one case, increased T2-STIR signal intensity remained unchanged in midsegments and increased in the apical segments at follow-up assessment; however, the presence of breathing artifacts located at mid and apical levels is the most likely explanation for this isolated finding. No LGE was detected immediately postrace or during follow-up CMR for any of the triathletes.

**TABLE 4:** Global and regional analysis of left ventricular strain and rotational mechanics.

Myocardial deformation parameters	Postrace	Follow-up	*P*-value^b^	*P*-value*	*P*-value^§^
Global radial strain, %	46 ± 7	51 ± 8	NS	−	−
Global longitudinal strain, %	−28 ± 9	−28 ± 5	NS	−	−
Global circumferential strain, %	−21 ± 4	−21 ± 3	NS	−	−
Basal radial strain, %	28 ± 7	47 ± 10	0.003	NS	<0.001
Mid-cavity radial strain, %	54 ± 12	57 ± 10	NS		
Apical radial strain, %	54 ± 6	49 ± 8	NS		
Basal longitudinal strain, %	−28 ± 12	−29 ± 5	NS	NS	NS
Mid-cavity longitudinal strain, %	−28 ± 7	−27 ± 5	NS		
Apical longitudinal strain, %	−27 ± 9	−27 ± 6	NS		
Basal circumferential strain, %	−20.6 ± 2.8	−20 ± 3	NS	NS	NS
Mid-cavity circumferential strain, %	−22.3 ± 2.8	−23 ± 2	NS		
Apical circumferential strain, %	−23.3 ± 3.5	−20 ± 3	NS		
Basal rotation, °	−2.4 ± 1.9	−1.8 ± 1.7	NS	−	−
Apical rotation, °	7.8 ± 2.2	2.8 ± 1.3	<0.001	−	−
Twist, °	10.2 ± 2.9	4.6 ± 2.2	0.009	−	−

^b^t-test post-race vs. follow-up; *ANOVA inter-slice comparison analysis at follow-up; ^§^ANOVA inter-slice comparison analysis postrace.

### Intra- and inter-observer variability

Good-to-excellent intra- and interobserver variabilities were observed for all the measurements, with an inter-rater correlation coefficient between 0.92 and 0.98 and an intra-rater correlation coefficient between 0.92 and 0.98.

## Discussion

In a small cohort of master triathletes, we demonstrated acute and reversible changes in ventricular function and myocardial tissue composition after a full-distance Ironman Triathlon competition. Immediately after an Ironman race, we observed the development of myocardial edema, but the regionality of myocardial edema distribution was noteworthy, mainly involving basal myocardial segments and yielding a relative apical sparing pattern. Notably, myocardial edema was associated with lower peak radial strain values with a similar pattern of regional distribution.

### Myocardial edema

Previous experimental studies inducing edema in different conditions, such as coronary sinus hypertension, hypoproteinemia, lymphatic obstruction, or crystalloid cardioplegic solution, showed that edema was not always associated with a reduction in myocardial contractility, probably depending on the different methods used (20). In an experimental model of myocardial ischemia-reperfusion, myofibrillar edema was a mechanism of postischemic regional dysfunction (21). Interestingly, in patients with ST-elevation acute myocardial infarction, the edematous peri-infarct myocardium had attenuated circumferential strain compared with the remote myocardium, and strain parameters improved after the resolution of myocardial edema (22). In the setting of sports cardiology, in a cohort of 20 recreational athletes undergoing CMR within 48 h after marathon completion, the presence of localized myocardial edema (with a per-segment myocardial T2-ratio increase of 11%) was associated with an impaired regional function (13). This finding contrasts with other studies documenting the absence of myocardial edema after a Triathlon race or a marathon (14, 23). These differences may be due to the choice of imaging modality used for edema detection. Similar to previous studies (13, 24), we used the T2-STIR sequence that remains one of the predominant CMR modes for assessment of myocardial edema, but not T2 mapping. Despite the two methods have usually good agreement in assessing myocardial edema (25), whole heart coverage is desirable. Tahir acquired only three short-axis slices for T2 and T1 mappings, covering only 20–30% of the myocardium (14), whereas we achieved a complete LV coverage, without any gap between slices (usually 10–13 short-axis slices). Then, in the study by Tahir, some myocardial regions of edema could have been missed. Moreover, the discrepancies in the results can also depend on the athlete’s fitness level. Gaudreault found a larger amount of edema in athletes with relatively lower fitness. Furthermore, the extent of exertion may be a component of the development of myocardial edema. In our study, the athletes performed an Ironmen competition that was much more intense and demanding than the effort made by the athletes enrolled in the other studies. Indeed, exercise duration and fitness level are key determinants in the development of postexercise LV dysfunction (26).

A possible explanation for the relative apical sparing pattern distribution of myocardial edema observed in our cohort in the postrace period includes regional differences in density of cardiac sympathetic fibers and age-related differential β-adrenoceptors expression (27–30). Future studies investigating regional differences in adrenoreceptor reserve or the process of release and reuptake of neurotransmitters in the athletic population may help to further clarify this hypothesis.

### Deformation imaging and rotational mechanics

The results of our study cannot ascertain the reason for the reduction in radial strain in the presence of myocardial edema. The complex three-dimensional architecture of the myocardium makes it much harder to explain the mechanisms underlying the observed transient reduction in cardiac contractility and to understand the role of adaptive or maladaptive response to strenuous exercise. As shown by MR diffusion tensor imaging, subepicardial fibers display an anticlockwise spiral pattern from inside to outside, and, with increasing distance from the epicardium, fiber orientation undergoes a continuous change in angulation with respect to an axis normal to the epicardium, thus the fibers are more circular at the middle layer, and more radial at the subendocardial portion of the myocardium (31). Therefore, there is no definite beginning or end of the contractile chain, but rather a fine three-dimensional meshwork inducing longitudinal and circumferential shortening, radial thinning, and shear strains (32, 33). In our study, beyond the reduction in the radial strain of the basal myocardial segments, we observed increased twisting and apical rotation in the apical LV segments, possibly representing a compensatory mechanism to preserve LV systolic function. Accordingly, there were no significant changes in global LV function between postrace and 30-day follow-up CMR scans. Several studies evaluated strain patterns in athletes after strenuous and prolonged exercise using echocardiography and CMR tagging techniques, yet delivering variable results. A recent study showed impaired LV global longitudinal strain (LVGLS) after a recreational ultramarathon at moderate altitude, in agreement with previous studies focusing on different types of exercise (34–36). Moreover, in the study of Champigneulle et al., the LVGLS reduction has been documented in athletes early after a speed ascent of the Montblanc (4,808 m), thus in the presence of gradual hypoxic conditions potentially worsening cardiac function (37). In the study of Jouffroy et al., in which athletes had run 80 km, LVGLS was already reduced at 53 km (early before the end of the race) and was found to be decreased in nearly 50% of athletes after race completion (36). However, other authors did not find any functional cardiac alteration after prolonged exercise. In the study of Hanssen et al. (28 males, age of 41 ± 5 years), no changes in radial, circumferential or longitudinal strain could be identified by CMR tagging early after an amateur marathon race (38). In another cohort of 14 male non-elite runners, aged 32 ± 10 years, who completed the 42.2-km London marathon, only limited changes in strain, rotation, and torsion in both the subendocardial and subepicardial layers of the LV wall could be detected by speckle tracking echocardiography.

Based on the observation of functional adaptive changes of the heart, also matching an increase in biomarkers of myocardial injury (39), the term postexercise cardiac fatigue has been coined. Cardiac fatigue refers to functional and humoral alterations occurring after prolonged and strenuous exercises, which are transient and indicative of cardiac distress (40). In the 30-day follow-up scan, we documented the full reversibility of acute changes in myocardial tissue and function seen in the early postrace period.

### Subclinical myocardial fibrosis

Beyond benign junctional pattern, LGE was negative in all of our master athletes. The prevalence and clinical significance of myocardial scarring in the athletic population are still debated (41, 42). Discordant findings may be due to various reasons, including limited sample size, training volume, age, number of races, frequency, intensity, time, and type of exercise, as well as individual susceptibility and genetic determinants of cardiac remodeling (43, 44). Moreover, gadolinium and myocardial washout might vary after strenuous effort because of acute modifications of blood volume or renal function. Interestingly, coronary artery calcifications have been associated with subclinical myocardial damage in athletes, thus recommending particular attention to cardiovascular risk stratification, mainly in veteran athletes (45).

### Study limitations

Our study has a few limitations that must be addressed. First, the sample size was small, yielding a larger margin of error and limiting our ability to identify outliers. Second, we enrolled a cohort of amateur master triathletes with a net prevalence of men. Thus, the results cannot be generalized to elite triathletes and the female gender. Third, we did not perform a basal CMR acquisition because of limited athlete’s availability and to avoid multiple administrations of gadolinium-based contrast agents in the same healthy subject; however, our objective was to assess the reversibility of any possible acute changes documented in the early postrace period. Fourth, we acknowledge the lack of important pieces of information such as 12-lead ECG, levels of cardiac biomarkers (cardiac troponin, natriuretic peptides), and detailed data about the frequency, intensity, time, and type of training that led up to the competition. Finally, tissue characterization was limited to conventional T2-weighted STIR [with its intrinsic technical limitations (46), including low signal-to-noise ratio, high dependency on magnetic field inhomogeneity, susceptibility to motion artifacts, and loss of signal secondary to cardiac motion across the plane in black blood preparation, subendocardial slow flow hyperintensity] and LGE sequences. Parametric T1 and T2 mapping and extracellular volume fraction data were not available at the time of enrollment.

## Conclusion

The present study demonstrated significant early postrace changes in ventricular function and myocardial tissue composition and the full reversibility of these changes in a cohort of master Ironman triathletes. The results of this study add new insights regarding the acute cardiac response to strenuous and prolonged exercise on myocardial tissue component, function, and the reversible nature of these changes. In view of the variability of published results, larger multicenter studies are needed to confirm the presence, magnitude, and reversibility of myocardial functional and structural changes in order to help clarify the concept of cardiac fatigue and investigate the true prevalence of irreversible cardiac injury linked to intense and prolonged exercise.

## Data availability statement

The raw data supporting the conclusions of this article will be made available by the authors, without undue reservation.

## Ethics statement

This study involved human participants and has been reviewed and approved by the local Ethics Committee (Comitato Etico Locale dell’Azienda Ospedaliera Universitaria Pisana) of Pisa, Italy.

## Author contributions

AP: full access to all the data in the study and took responsibility for the integrity of the data and the accuracy of the data analysis. FR, GA, and AP: study concept and design. FR, GA, AP, CM, and SR: acquisition, analysis, or interpretation of data. FR, AP, and CD: drafting of the manuscript. FR: statistical analysis. GA: administrative, technical, or material support. SG and AP: study supervision. All authors critical revision of the manuscript for important intellectual content and approved the submitted version.

## Footnotes

1
www.R-project.org
